# Brown Adipose Tissue Is Linked to a Distinct Thermoregulatory Response to Mild Cold in People

**DOI:** 10.3389/fphys.2016.00129

**Published:** 2016-04-19

**Authors:** Maria Chondronikola, Elena Volpi, Elisabet Børsheim, Tony Chao, Craig Porter, Palam Annamalai, Christina Yfanti, Sebastien M. Labbe, Nicholas M. Hurren, Ioannis Malagaris, Fernardo Cesani, Labros S. Sidossis

**Affiliations:** ^1^Metabolism Unit, Shriners Hospitals for Children-GalvestonTX, USA; ^2^Department of Preventive Medicine and Community Health, University of Texas Medical BranchGalveston, TX, USA; ^3^Division of Rehabilitation Sciences, Department of Nutrition and Metabolism, University of Texas Medical BranchGalveston, TX, USA; ^4^Department of Nutrition and Dietetics, Harokopio University of AthensGreece; ^5^Institute for Translational Sciences, University of Texas Medical BranchGalveston, TX, USA; ^6^Sealy Center on Aging, University of Texas Medical BranchGalveston, TX, USA; ^7^Department of Internal Medicine, University of Texas Medical BranchGalveston, TX, USA; ^8^Department of Surgery, University of Texas Medical BranchGalveston, TX, USA; ^9^Department of Interventional Radiology, University of Texas Medical BranchGalveston, TX, USA; ^10^Quebec Heart and Lung Research Institute CentreQuebec, QC, Canada; ^11^Department of Nuclear Medicine, University of Texas Medical BranchGalveston, TX, USA

**Keywords:** brown adipose tissue, thermoregulation, body core temperature, supraclavicular skin temperature, cold exposure

## Abstract

Brown adipose tissue (BAT) plays an important role in thermoregulation in rodents. Its role in temperature homeostasis in people is less studied. To this end, we recruited 18 men [8 subjects with no/minimal BAT activity (BAT−) and 10 with pronounced BAT activity (BAT+)]. Each volunteer participated in a 6 h, individualized, non-shivering cold exposure protocol. BAT was quantified using positron emission tomography/computed tomography. Body core and skin temperatures were measured using a telemetric pill and wireless thermistors, respectively. Core body temperature decreased during cold exposure in the BAT− group only (−0.34°C, 95% CI: −0.6 to −0.1, *p* = 0.03), while the cold-induced change in core temperature was significantly different between BAT+ and BAT− subjects (BAT+ vs. BAT−, 0.43°C, 95% CI: 0.20–0.65, *p* = 0.0014). BAT volume was associated with the cold-induced change in core temperature (*p* = 0.01) even after adjustment for age and adiposity. Compared to the BAT− group, BAT+ subjects tolerated a lower ambient temperature (BAT−: 20.6 ± 0.3°C vs. BAT+: 19.8 ± 0.3°C, *p* = 0.035) without shivering. The cold-induced change in core temperature (*r* = 0.79, *p* = 0.001) and supraclavicular temperature (*r* = 0.58, *p* = 0.014) correlated with BAT volume, suggesting that these non-invasive measures can be potentially used as surrogate markers of BAT when other methods to detect BAT are not available or their use is not warranted. These results demonstrate a physiologically significant role for BAT in thermoregulation in people. This trial has been registered with Clinaltrials.gov: NCT01791114 (https://clinicaltrials.gov/ct2/show/NCT01791114).

## Introduction

Thermoregulation is a vital homeostatic mechanism maintaining the core body temperature within a relatively narrow range in the face of large fluctuations in ambient temperature (Mekjavic and Eiken, 2006). Deviation from this normal range may indicate the presence of a pathological condition and can be lethal in extreme cases. Brown adipose tissue (BAT) has been shown to be the primary thermoregulatory tissue during non-shivering cold exposure (CE) in mammals (Cannon and Nedergaard, 2004). Its thermogenic properties are attributable to its numerous mitochondria, which contain high amounts of the uncoupling protein 1 (UCP1, also known as *thermogenin*) (Nedergaard et al., 2001). UCP1 uncouples oxidative phosphorylation, resulting in heat production (thermogenesis).

BAT has only recently been identified in adults (Nedergaard et al., 2007; Cypess et al., 2009; van Marken Lichtenbelt et al., 2009; Virtanen et al., 2009) and thus its thermoregulatory role in people remains unclear. Historically, BAT has been proposed to play a role in thermoregulation in infants, who have copious amounts of BAT (Ito and Kuroshima, 1967), apparently because they lack the ability to shiver (Silverman et al., 1964; Dawkins and Scopes, 1965). Acute CE (2–6 h) activates BAT (van Marken Lichtenbelt et al., 2009; Virtanen et al., 2009; Ouellet et al., 2012; Chondronikola et al., 2014), while chronic CE (10 days to 6 weeks) can further increase the detectable BAT activity in lean, obese, and patients with type 2 diabetes (Yoneshiro et al., 2013; van der Lans et al., 2013; Blondin et al., 2014; Lee et al., 2014; Hanssen et al., 2015a,b). Consistent with results of the classic study by Davis (1961) more than 50 years earlier, it has been recently reported that chronic CE not only increases BAT activity, but also increases thermal comfort and trunk skin temperature (van der Lans et al., 2013; Hanssen et al., 2015a,b). However, the magnitude of change in the previously mentioned responses was not correlated with BAT. Blondin et al. (2014) also reported an increase in BAT activity after 4-week cold acclimation, but no difference in the thermal responses of subjects before and after acclimation. Therefore, the physiological significance of BAT in human thermoregulation remains unclear.

Considering the lack of evidence on the thermoregulatory role of human BAT, we conducted a study to determine if there is a physiologically significant role of BAT in temperature homeostasis in people. We studied 18 men with significant BAT activity (BAT+; *n* = 10) or without/minimal BAT activity (BAT−; *n* = 8), using an individualized, 6 h, non-shivering CE protocol. We found that the presence of BAT is associated with higher tolerance to cold, supported by the findings of higher core body temperature during CE noted in subjects with high amounts of BAT, while the BAT+ group was able to tolerate a lower ambient temperature without shivering. These results support a physiologically significant role for human BAT in thermoregulation.

## Materials and methods

### Participants

**Figure 1:**
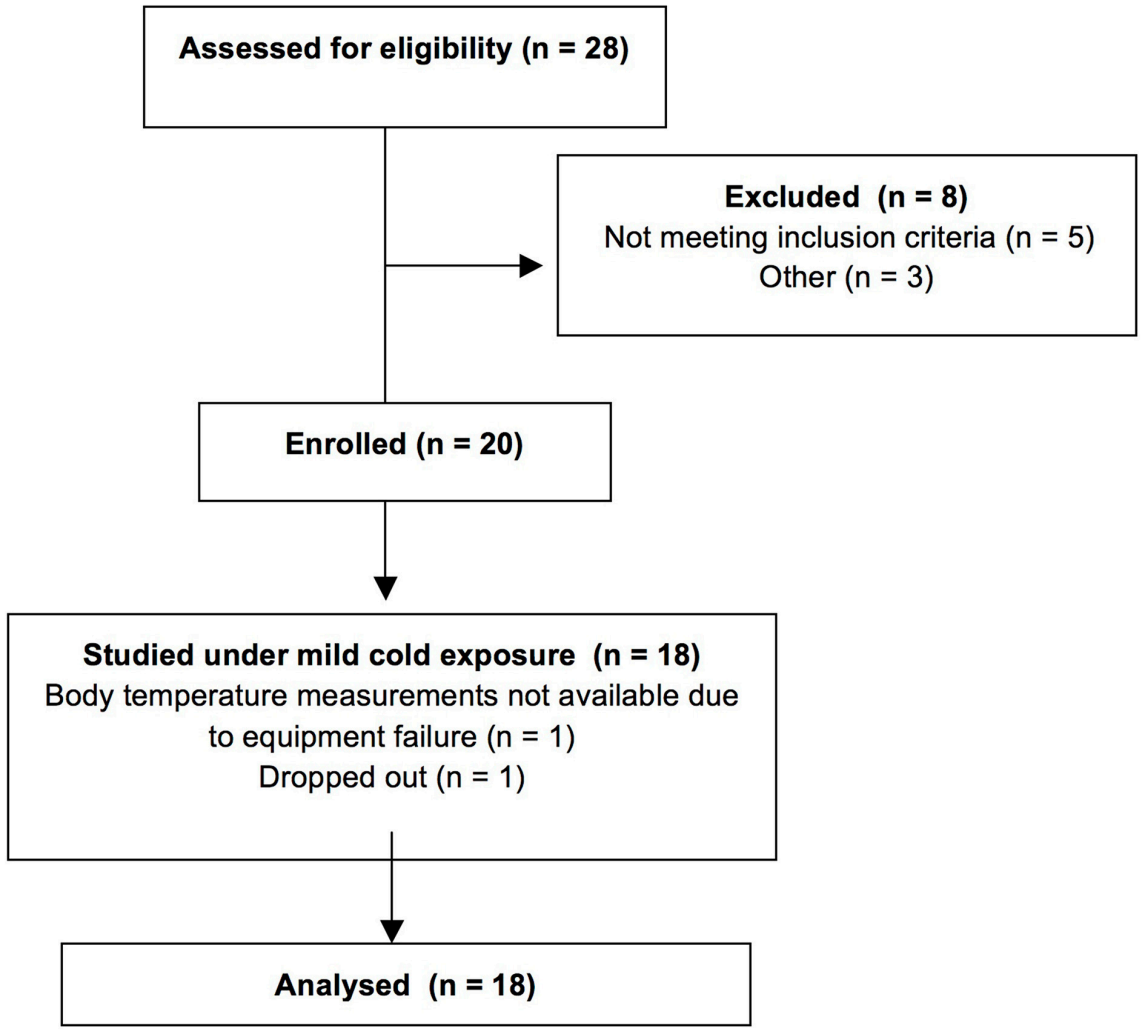
**CONSORT diagram of the study**.

Twenty men enrolled in this study. Only healthy subjects qualified to participate. Informed written consent was obtained from all participants in accordance with the Declaration of Helsinki; the University of Texas Medical Branch Institutional Review Board and the Institute for Translational Sciences (ITS) Scientific Review Committee approved the study protocol. From the subjects enrolled in the study, one participant dropped out, while body temperature data were not recorded for one participant due to equipment failure. Results from 18 participants were analyzed (Figure 1). This trial has been registered with Clinaltrials.gov: NCT01791114 https://clinicaltrials.gov/ct2/show/NCT01791114).

### Experimental protocol

Three days prior to the study, participants were asked to follow their regular weight-maintaining diet and to refrain from any excessive physical activity, alcohol, and caffeine consumption. The evening before the study, the subjects were admitted to the ITS Clinical Research Center and offered a standardized meal. Subjects fasted and rested in bed overnight, wearing standardized clothing (a T-shirt and a pair of shorts), and covered with a blanket. The temperature of the room was 23–24°C.

The following morning a 6 h, individualized, CE protocol was employed to maximally induce non-shivering thermogenesis. In addition to the standardized clothing, subjects wore garments cooled by liquid circulation (Cool Flow® vest and blanket and Arctic Chiller cooling system, Polar Products Inc., Stow, OH) and laid supine. The temperatures of the cooling garments and the room were initially set at approximately 19–20°C and were decreased by 1°C approximately every 30 min until subjects reported shivering. Then, the cooling garment and ambient temperatures were increased by 1°C and adjusted as needed to prevent shivering. Additionally, we visually inspected the subjects for shivering.

### Temperature measurements and thermal sensation

Wireless probes (iButtons, Maxim, Dallas, TX) were used to measure the skin temperature of the participants, the room, and the water circulating through the cooling garments. The probes were placed using adhesive tape in 14 locations of the body (forehead, neck, right scapula, left upper chest, right arm in the upper location, left arm in the lower location, left hand, right abdomen, left paravertebral area, right anterior thigh, left posterior thigh, right shin, left calf, and right instep) according to a standard protocol (ISO9886, 2004). The average skin temperature was calculated as the average of those 14 probes (ISO9886, 2004). One additional probe was placed on the skin area over the left supraclavicular adipose tissue depot (where BAT is usually localized). Temperature recording using the wireless thermistors failed in two participants.

The trunk temperature was calculated as the average of the skin temperatures recorded by the probes placed in the right scapula, the paravertebral area, the abdomen, and the chest, while the distal temperature was calculated as the average skin temperatures of the probes placed on the instep and hand (Wijers et al., 2011). The temperature gradient between the foot and the ambient temperature was used as an index of vasoconstriction and skin perfusion (Ruiz et al., 1979). Thermal sensation was assessed using a visual analog scale of the American Society of Heating, Refrigerating, and Air-Conditioning Engineers (de Dear et al., 1998; de Deara and Brager, 2002). Core temperatures were measured using a telemetric pill (Core-Temp, HQ Inc., Palmeto, FL) that was ingested by 14 participants (6 BAT− and 8 BAT+).

### Positron emission tomography/computerized tomography (PET/CT)

After 5 h of CE, subjects were given a bolus injection of 185 MBq of 2-deoxy-2-(^18^F)fluoro-D-glucose (^18^F-FDG). One hour later, a PET/CT (General Electric Medical Systems, Milwaukee, WI) scan was performed to assess BAT volume (ml) and mean standardized uptake value (SUV; g/ml). We assessed the PET/CT scans for ^18^F-FDG BAT using the following criteria: (a) ^18^F-FDG uptake was evident in the cervical/supraclavicular, mediastinal, paravertebral, and/or perirenal areas; (b) ^18^F-FDG uptake had a mean SUV of 1.5 or greater (an indicator of ^18^F-FDG uptake intensity); and (c) the tissue corresponded to the density of adipose tissue on CT (−190 to −30 Hounsfield units). The mean SUVs for each identified deposit were determined using commercial fusion software (MIM software; MIMvista Corp., Cleveland, OH). The volume of ^18^F-FDG BAT was quantified by autocontouring each identified individual BAT deposit (with a SUV ≥ 1.5).

### Body composition

We used Dual X-Ray Absorptiometry to evaluate the lean body mass and total body fat mass of the participants (Hologic model QDR-4500W, Hologic Inc., Bedford, MA).

### Statistical analysis

All results are presented as means ± standard deviations. BAT+ and BAT− subjects were compared using Student's *t*-test (for normally distributed data) or the Mann Whitney test (for not normally distributed data). The one sample *t*-test was used to compare if the cold-induced change in body core and supraclavicular temperatures was different from 0 in BAT+ and BAT− participants. Paired *t*-test (for normally distributed data) or Wilcoxon singed-rank test (for not normally distributed data) were used to compare CE and TN conditions. The Pearson's r was used to evaluate the correlation between BAT or muscle activity with body temperatures. A multiple linear regression modeled the relation between each outcome and BAT volume, while adjusting for the potentially prognostic covariates age and percent fat. BAT volume was log (base 10) transformed for better centering and interpretation. Statistical analyses were performed using Graph Pad version 5 for Mac OS X (Graph Pad Software, Inc. La Jolla, CA) and SPSS Version 20 for Mac statistical software (IBM Inc., Armonk, NY). All statistical tests assumed a 95% level of confidence.

## Results

### Subject characteristics

The study sample consisted of 18 men (Table 1). Participants were divided into two groups: 10 subjects with significant BAT activity (BAT+) and 8 subjects with no/minimal BAT activity (BAT−). The two groups were significantly different, by design, in BAT volume [63.1 ml, 95% confidence interval (CI): 31.8–94.4, *p* = 0.001] and activity (1.3 g/ml, 95% CI: 0.7–2.0, *p* = 0.001). Moreover, the participants in the BAT− group were older (19.1 years, 95% CI: 3–35, *p* = 0.02) and tended to have higher body fat content (7.8%, 95% CI: −0.9 to 16.5, *p* = 0.08) compared to the BAT+ group.

**Table 1:** **Subject characteristics**.

**Parameters**	**All (*n* = 18)**	**BAT− (*n* = 8)**	**BAT+ (*n* = 10)**
Age (y)	46.9 ± 18.1	57.5 ± 16.2	38.4 ± 15.3^*^
BMI (kg/m^2^)	29.5 ± 4.6	31.0 ± 3.2	28.2 ± 5.4
BSA (m^2^)	2.05 ± 0.15	2.05 ± 0.12	2.05 ± 0.19
Lean Body Mass (kg)	61.0 ± 8.0	59.6 ± 5.6	62.2 ± 9.7
Body Fat (%)	31.7 ± 9.3	36.0 ± 3.4	28.2 ± 11.1
BAT volume (ml)	38.7 ± 44.2	3.7 ± 4.7	66.9 ± 41.3^***^
BAT mean SUV (g/ml)	1.75 ± 0.91	1.03 ± 0.73	2.34 ± 0.57^***^

Data are mean standard deviations.

* p = 0.02

*** p = 0.001

by Student's t-test between BAT+ and BAT− subjects. BMI, body mass index; BAT, brown adipose tissue; BAT+, detectable BAT; BAT−, no detectable BAT; BSA: SUV, standard uptake value.

### Effect of BAT activation on tolerance to CE and thermal sensation

**Figure 2:**
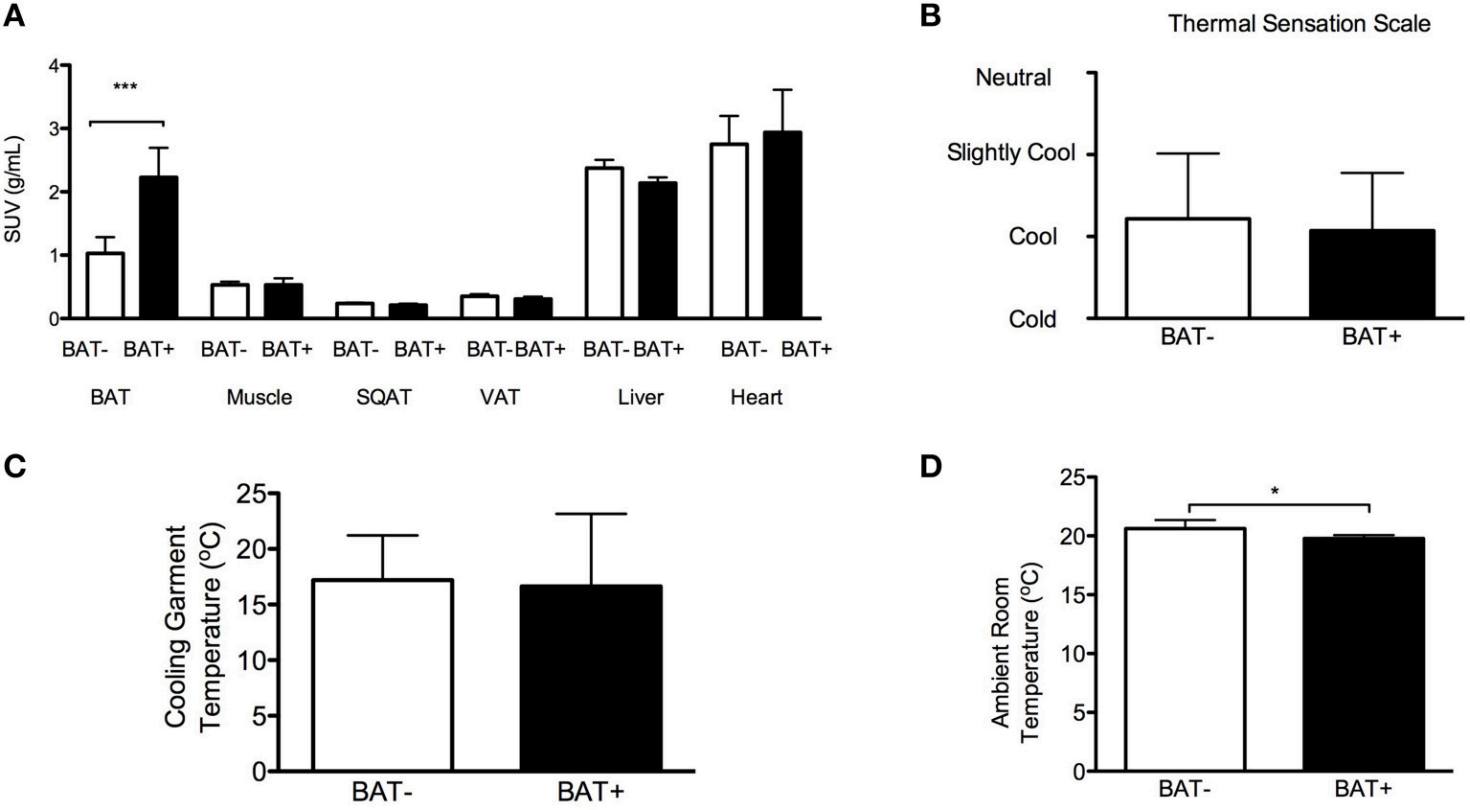
**Brown adipose tissue (BAT), cold exposure (CE) tolerance, and thermal sensation. (A)** Mean standardized uptake value (SUV) for glucose in various tissues at 6 h of CE. SQAT, subcutaneous adipose tissue; VAT, visceral adipose tissue. **(B)** Thermal sensation in subjects with detectable BAT (BAT+) and without detectable BAT (BAT−) at 5 h of CE. **(C)** Cooling garments temperature in BAT+ and BAT− subjects at 5 h of CE. **(D)** Ambient room temperature in BAT+ and BAT− subjects at 5 h of CE. The data are means and standard deviations. ^*^p = 0.035 using Mann–Whitney test and ^***^p = 0.001 using paired *t*-test.

The study participants followed an individualized CE protocol to maximally induce non-shivering thermogenesis (i.e., ambient and garment water temperatures were adjusted to the lowest level tolerated by each subject without shivering). As expected, the results of the ^18^F-FDG-PET/CT analyses support that the BAT+ group demonstrated higher BAT metabolic activity compared to BAT− subjects. No significant differences were observed in the other tissues between BAT+ and BAT− subjects (Figure 2A).

BAT+ and BAT− subjects reported comparable levels of thermal sensation during CE (Figure 2B). Subjectively, both groups reported “feeling cool” during the study. According to the study protocol, the temperature of the room, and the cooling garments was titrated to the lower tolerable level without shivering. The temperature of the water circulating in the cooling garments was also similar in the two groups (Figure 2C). However, the room temperature was slightly lower in the BAT+ group (BAT−: 20.6 ± 0.3°C vs. BAT+: 19.8 ± 0.3°C, *p* = 0.035, Figure 2D). These findings suggest that the subjects in the BAT+ group had a higher tolerance to cold.

### Effect of BAT activation on body core temperature

**Figure 3:**
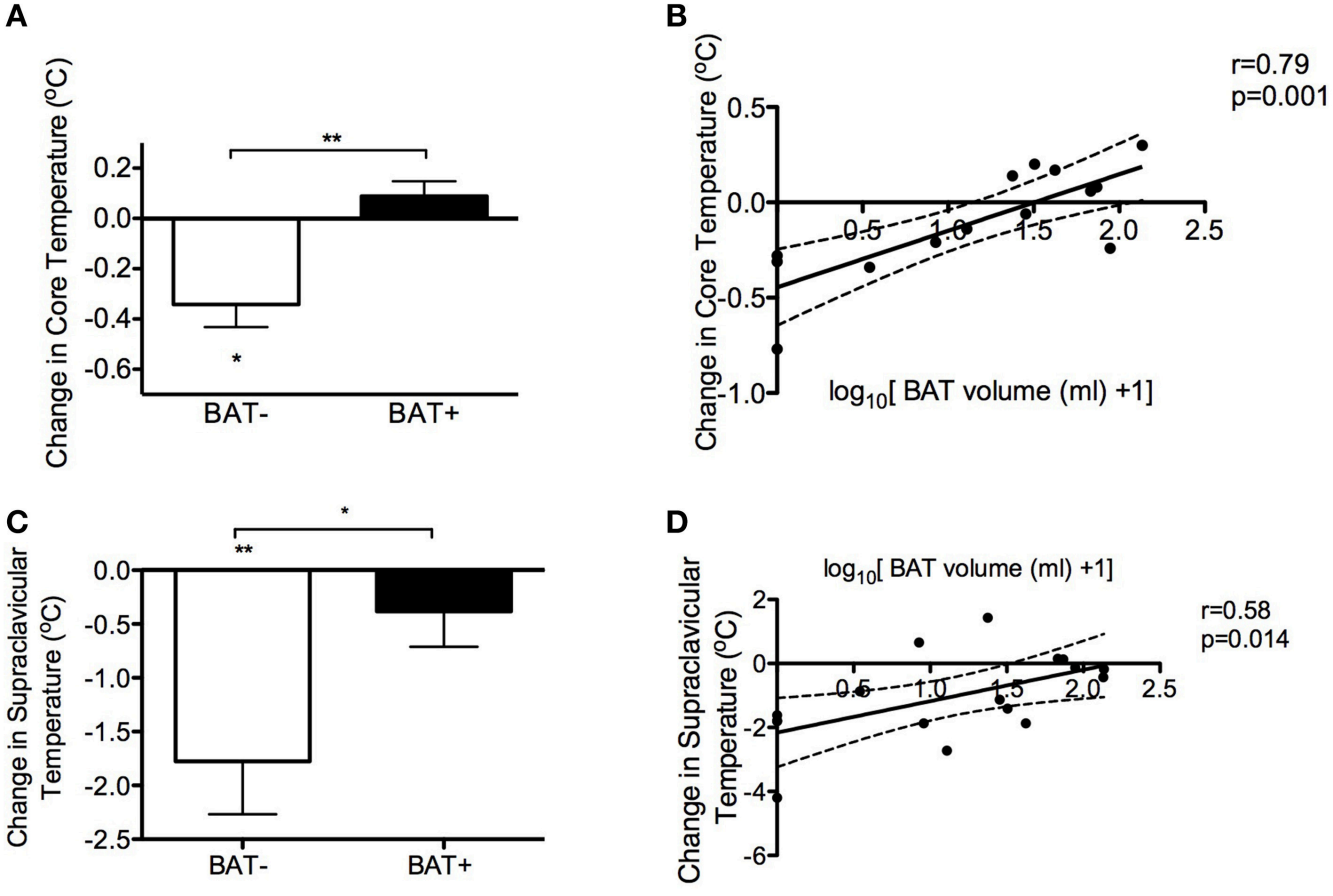
**Brown adipose tissue (BAT) and body temperature. (A)** Cold-induced change in core temperature in subjects with detectable BAT (BAT+) and without detectable BAT (BAT−). **p = 0.0014 using Student's *t*-test, ^*^p = 0.03 using one sample *t*-test. **(B)** Correlation of BAT volume with the change in body core temperature using Pearson's r. **(C)** Cold-induced change in supraclavicular skin temperature in BAT+ and BAT− subjects. ^**^p = 0.007 using by one sample *t*-test, ^*^p = 0.03 using Student's *t*-test. **(D)** Correlation of BAT volume with the change in supraclavicular skin body temperature using Pearson's r. The data are means and standard deviations. The dashed lines represent 95% confidence intervals.

Core body temperature decreased after 5 h of CE only in the BAT− group (−0.34°C, 95% CI: −0.6 to −0.1, *p* = 0.03) (Figure 3A). Interestingly, the cold-induced change in core temperature was significantly different between BAT+ and BAT− subjects (0.43°C, 95% CI: 0.20–0.65, *p* = 0.0014), while BAT volume significantly correlated with cold-induced change in core temperature (*r* = 0.79, *p* = 0.001, Figure 3B). No correlation was noted between muscle activity (measured as the mean skeletal muscle SUV for glucose during CE in the *m*. *pectoralis major* and *m*. *vastus lateralis*) and change in core temperature at the 5 h of CE (Table 2) or any other time point (data not shown).

**Table 2:** **Muscle metabolic activity and body temperatures**.

**Parameters**	
Change in core temperature (°C)	*r* = −0.324
	*p* = 0.259
Change in supraclavicular skin temperature (°C)	*r* = −0.412
	*p* = 0.101
Change in trunk temperature (°C)	*r* = 0.103
	*p* = 0.685

The correlation coefficients were calculated using Pearson's r.

BAT activity has been inversely associated with age (Cypess et al., 2009; Yoneshiro et al., 2011) and adiposity (Saito et al., 2009; van Marken Lichtenbelt et al., 2009). To account for potential confounding, we performed multiple linear regression analysis adjusting for age and adiposity (Table 3). After adjustment for age and adiposity, BAT volume was significantly associated with higher body core temperature (*p* = 0.01) after 5 h of CE. Collectively, these results suggest that cold-stimulated BAT activity can be involved in core temperature regulation in people.

### Effect of BAT activation on skin temperature

We further hypothesized that, should BAT have a thermoregulatory role in people, the skin temperature of the BAT+ subjects would have a different response to cold than that of BAT− subjects, especially in areas adjacent to the anatomical locations of BAT (i.e., supraclavicular, peri-renal, mediastinal, and paravertebral areas). CE decreased the skin temperature of the supraclavicular area in the BAT− group only (−1.8°C, 95% CI: −2.9 to −0.6, *p* = 0.007, Figure 3C), while no significant change was noted in the BAT+ group. The cold-induced change in the supraclavicular skin temperature was significantly different between BAT+ and BAT− subjects (−1.4°C, 95% CI: −2.6 to −0.2, *p* = 0.03). The cold-induced change in supraclavicular temperature (*r* = 0.58, *p* = 0.014) correlated with BAT volume (Figure 3D). No correlation was noted between muscle activity and the supraclavicular or trunk skin temperature during the last hour of CE (Table 2). Finally, BAT volume was significantly associated with supraclavicular (*p* = 0.03) and marginally associated with trunk skin temperature (*p* = 0.07) after adjustment for age and adiposity (Table 3). These data further demonstrate a significant role for human BAT in thermoregulation, where cold-induced BAT activation affects skin temperature in areas adjacent to the anatomical localization of BAT.

**Table 3:** **Multiple linear regression analysis**.

**Independent predictors**	**Univariate analysis**				**Multiple regression analysis**			
	**Beta**	**St. Error**	**St. Beta**	***p*-value**	**Beta**	**St. Error**	**St. Beta**	***p*-value**
**DEPENDENT VARIABLE: CHANGE IN CORE TEMPERATURE DURING THE 5 h OF COLD EXPOSURE**								
BAT volume^a^	0.244	0.056	0.784	0.001	0.254	0.08	0.79	0.01
Body fat %	−0.013	0.009	−0.398	0.159	−0.005	0.007	−0.15	0.534
Age	−0.008	0.004	−0.481	0.08	0.002	0.005	0.13	0.641
**DEPENDENT VARIABLE: CHANGE IN SUPRACLAVICULAR TEMPERATURE DURING THE 5 h OF COLD EXPOSURE**								
BAT volume^a^	0.870	0.301	0.599	0.011	1.048	0.430	0.721	0.030
Body fat %	−0.058	0.037	−0.373	0.140	−0.029	0.040	−0.191	0.475
Age	−0.021	0.019	−0.278	0.279	0.024	0.023	0.319	0.319
**DEPENDENT VARIABLE: CHANGE IN TRUNK TEMPERATURE DURING THE 5 h OF COLD EXPOSURE**								
BAT volume^a^	0.570	0.413	0.326	0.187	0.973	0.501	0.557	0.070
Body fat %	−0.67	0.040	−0.388	0.112	−0.094	0.045	−0.544	0.057
Age	0.002	0.022	0.023	0.927	0.066	0.028	0.742	0.032

a BAT volume was transformed to log_10_[BAT volume (ml) + 1].

### Body temperatures as non-invasive indices of BAT activity

Since the available methods to estimate BAT volume [e.g., PET/CT, magnetic resonance imaging (MRI), or infrared thermography] involve exposure to radiation and/or are costly and labor-intensive, we propose that changes in body core and skin temperatures during CE can be potentially used as surrogate markers of BAT activity. To further validate those indices as surrogate markers, we tested their correlation with BAT activity for the different durations of CE (Table 4). Change in core and supraclavicular temperatures were significantly correlated with BAT activity after 3–4 h of CE, respectively.

**Table 4:** **Indexes of brown adipose tissue volume and duration of cold exposure**.

**Parameters**	**1 h**	**2 h**	**3 h**	**4 h**	**5 h**
Change in core temperature (°C)	*r* = 0.34	*r* = 0.37	***r*** = **0.63**	***r*** = **0.67**	***r*** = **0.79**
	*p* = 0.24	*p* = 0.20	***p*** = **0.016**	***p*** = **0.012**	***p*** = **0.001**
Change in supraclavicular skin temperature (°C)	*r* = 0.42	*r* = 0.42	*r* = 0.45	***r*** = **0.56**	***r*** = **0.584**
	*p* = 0.1	*p* = 0.1	*p* = 0.1	***p*** = **0.019**	***p*** = **0.014**

BAT volume was transformed to log_10_[BAT volume (ml) +1]. The correlation coefficients were calculated using Pearson's r. The bold values indicate statistically significant correlations.

### BAT activation and cardiovascular function in response to CE

**Figure 4:**
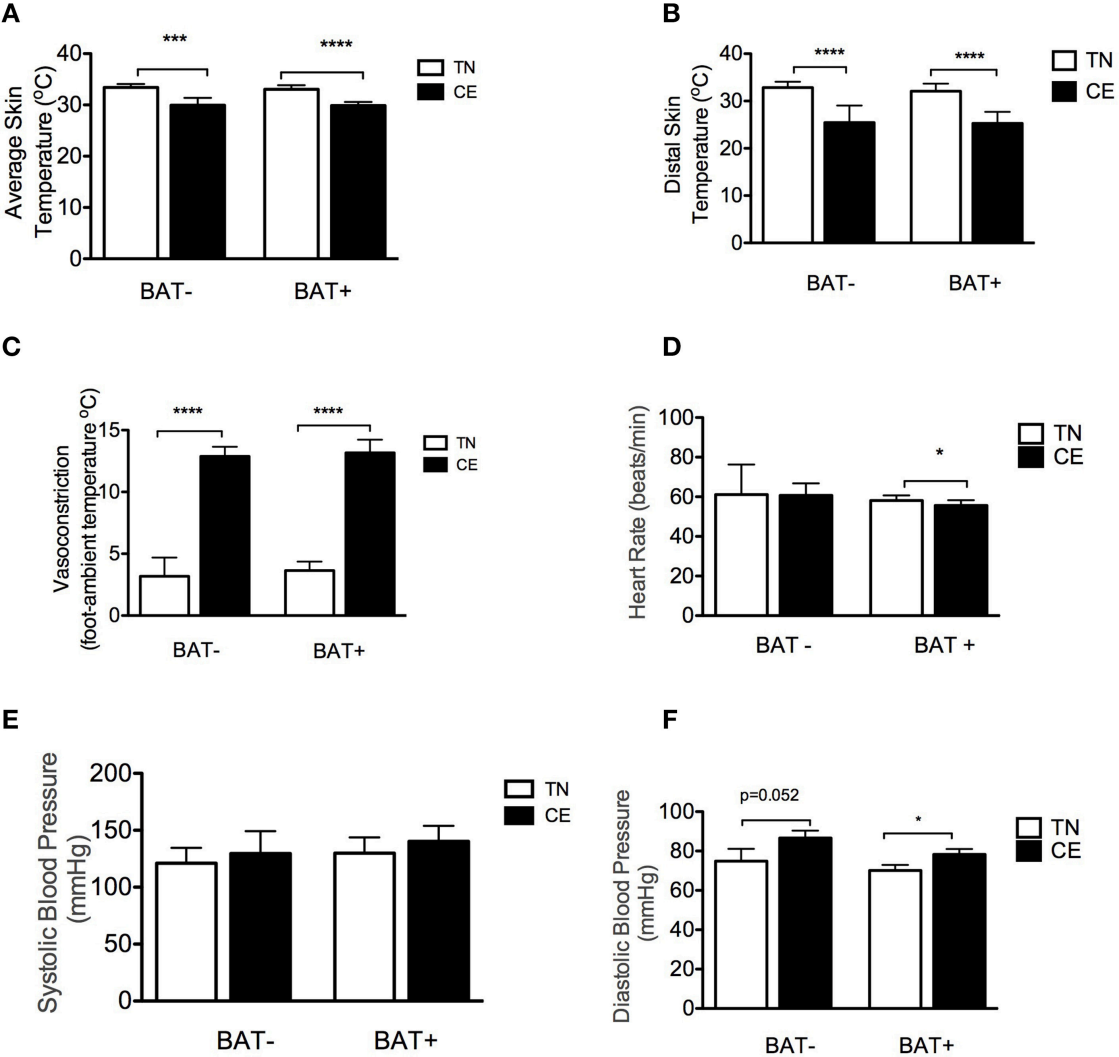
**Brown adipose tissue (BAT) activation, skin perfusion, and cardiovascular response to cold exposure (CE). (A)** Average skin temperature in subjects with detectable BAT (BAT+) and without detectable BAT (BAT−) in thermoneutral (TN) conditions and at 5 h of CE. **(B)** Distal (hand, foot) skin temperature in BAT+ and BAT− subjects in TN conditions and after 5 h of CE. **(C)** Vasoconstriction/skin perfusion in BAT+ and BAT− subjects in TN conditions and at 5 h of CE. **(D)** Heart rate in BAT+ and BAT− subjects in TN conditions and at 5 h of CE. **(E,F)** Systolic **(E)** and diastolic **(F)** blood pressure in BAT+ and BAT− subjects in TN conditions and at 5 h of CE. Data are means and standard deviations. The data are means and SD. ^*^p < 0.05, ^***^p = 0.001, ^****^p < 0.001 using paired *t*-test.

Finally, we investigated the cold-induced changes in markers of cardiovascular function and cutaneous perfusion in BAT+ and BAT− subjects. The average skin temperature (BAT−: −3.5°C, 95% CI: −4.8 to −2.1, *p* = 0.001 and BAT+: −3.2°C, 95% CI: −3.7 to −2.7, *p* < 0.001, Figure 4A) and distal skin temperature (BAT−: −7.4°C, 95% CI: −5.1 to −9.7, *p* < 0.001 and BAT+: −6.8°C, 95% CI: −8.9 to −4.8, *p* < 0.001, Figure 4B) significantly decreased in both groups. Moreover, both groups displayed a similar degree of peripheral vasoconstriction during CE (BAT−: −9.9°C, 95% CI: −11.0 to −7.9, *p* < 0.001 and BAT+: −9.5°C, 95% CI: −12.2 to −6.9, *p* < 0.001, Figure 4C). Additionally, CE significantly decreased heart rate only in the BAT+ group (−2.5 beats/min, 95% CI: −4.7 to −0.3 *p* = 0.03, Figure 4D). We noted no significant differences in systolic blood pressure (Figure 4E), while diastolic blood pressure increased in both groups (BAT−: 11.75 mmHg, 95% CI: 0.1–23.6, *p* = 0.052 and BAT+: 8.2 mmHg, 95% CI: 1.0–15.4, *p* = 0.03, Figure 4F).

## Discussion

The results of this study provide evidence of the physiological role of BAT in thermoregulation in people. BAT volume was a significant predictor of the cold-induced change in core temperature, adding to the notion that BAT activation contributes to homeothermy. Moreover, having significant amounts of detectable BAT was associated with increased capacity to compensate for heat loss, as evidenced by the lower ambient CE temperature tolerated in the BAT+ group without shivering. Finally, the significant correlation between the cold-induced change in core and supraclavicular temperature suggest those two measures as a potential surrogate markers of BAT activity.

The first line of evidence suggesting a physiological role of BAT in thermoregulation is the finding that the subjects in the BAT+ group demonstrated different response in their core body temperature after 5 h of non-shivering CE compared to BAT− subjects. Namely, core body temperature significantly decreased in the BAT− group, but it remained unchanged in the BAT+ group. BAT is strategically localized in close proximity to central vessels (carotid artery, brachiocephalic artery, epicardial coronary artery, cardiac veins, and others) (Sacks and Symonds, 2013), which increases the efficiency of heat transfer to core organs.

We did not find any significant differences between the two groups in the indices used to evaluate vasoconstriction/cutaneous skin perfusion. Heart rate decreased significantly in BAT+ subjects during CE, while systolic blood pressure was significantly elevated in the BAT+ group and marginally elevated in BAT− subjects. These results suggest that BAT+ and BAT− subjects may have distinct cardiovascular responses to CE.

Maintenance of core body temperature within a narrow range, despite the influence of external stimuli, is of vital importance to mammals (Mekjavic and Eiken, 2006). Early studies in rodents demonstrated that BAT temperature increases during CE, providing direct evidence for the thermoregulatory role of BAT in mammals (Donhoffer et al., 1964). In the current study, the intensity of CE, which was higher in the BAT+ group, was titrated individually to induce maximal non-shivering thermogenesis. The small decrease in core temperature noted in the BAT− participants indicates the decreased ability of this group to keep the core temperature constant during prolonged CE. Should this decrease in body core temperature have continued, it would likely have resulted in shivering to prevent further decline. The magnitude of change in core temperature was strongly correlated with BAT volume, while the association of BAT with core temperature remained statistically significant after adjustment for age and adiposity. Muscle metabolic activity was not significantly correlated with body core temperature.

Having established a role for BAT in thermoregulation in adult humans, we tested the ability of core temperature and skin temperatures to predict BAT volume. The methods currently available to measure BAT volume and activity (PET/CT, MRI, infrared thermography, etc.) are expensive and involve exposure to radiation and/or require availability of specialized equipment. Therefore, surrogate measures of BAT volume using methods that are safer, easy to perform, and less expensive than those outlined above, could become useful research tools. BAT is primarily located in the supraclavicular, paravertebral, and, perirenal areas. We hypothesized that skin temperatures adjacent to BAT may provide low-cost, non-invasive surrogate markers of BAT activity. Consistent with our hypothesis, BAT volume significantly correlated with changes in core and supraclavicular skin temperature. In addition, BAT volume was marginally associated with the change in trunk temperature after adjustment for age and adiposity. Studies in young lean subjects and children have proposed supraclavicular temperature (Symonds et al., 2012; Boon et al., 2014; van der Lans et al., 2016) and the gradient between the supraclavicular and the lateral upper chest temperatures (Jang et al., 2014) as surrogate measures of BAT activity. Here, we provide evidence that supraclavicular skin and core body temperatures can be used markers of BAT volume in a more diverse population when the other methods for the detection of BAT activity are not available or contraindicated.

Results from previous studies, in which BAT has not been measured support the link between temperature homeostasis with age and adiposity (Hayward and Keatinge, 1981; Kenney and Munce, 2003; Wijers et al., 2010). On the other hand, BAT activity has been inversely associated with age (Cypess et al., 2009; Yoneshiro et al., 2011) and adiposity (Saito et al., 2009; van Marken Lichtenbelt et al., 2009). Therefore, it is likely that BAT levels may at least partially explain this relationship between adiposity, aging, and temperature homeostasis. The results of this study have been statistically adjusted to account for a potential confounding effect of age and adiposity and they support the independent the role of BAT in thermoregulation in humans. Moreover, when we restricted our groups to subjects matched for age and adiposity the reported outcomes between the two groups remained the same further supporting our conclusions (data not shown).

Our results support the notion that BAT plays a role in thermoregulation by increasing heat production. BAT and adiposity are inversely correlated (van Marken Lichtenbelt et al., 2009). Furthermore, weight loss (Vijgen et al., 2012; Orava et al., 2013) and chronic CE (Yoneshiro et al., 2013) increase BAT and decrease body fat. We could thus speculate that a chronic positive energy balance that increases adiposity may lead to greater insulation and, potentially, underutilization of BAT for heat production. This will ultimately result in further weight gain and its related metabolic abnormalities (hyperlipidemia, insulin resistance, etc.).

Thermoregulation constitutes an important homeostatic mechanism, tightly linked to survival. This study provides evidence for the physiologically significant role of BAT in thermoregulation in people. Moreover, we propose two indices that can be used to estimate BAT volume when radiological approaches or other techniques are not available, and/or their use is contraindicated. Further research is needed to understand the role of human BAT in pathological conditions that cause a shift in core body temperature from the null zone (fever, anesthesia) and conditions that affect heat loss or production (e.g., burn injury).

## Author contributions

Conception and design of the work: LS, EV, EB, MC, CP, and CY. Acquisition, analysis, or interpretation of data for the work: MC, EV, EB, TC, CP, PA, CY, SL, NH, IM, FC, and LS. Drafting of the work or revising it critically for important intellectual content: MC, EV, EB, TC, CP, PA, CY, SL, NH, IM, FC, and LS. All authors have approved the final version of the manuscript and agree to be accountable for all aspects of the work in ensuring that questions related to the accuracy or integrity of any part of the work are appropriately investigated and resolved. All persons designated as authors qualify for authorship, and all those who qualify for authorship are listed.

## Funding

This study was conducted with the support of the Institute for Translational Sciences at the University of Texas Medical Branch, supported in part by a Clinical and Translational Science Award (UL1TR000071) from the National Center for Advancing Translational Sciences, National Institutes of Health, the American Diabetes Association (1-14-TS-35 to LS), Shriners Hospitals for Children grants (84090 and 85310 to LS), the John Sealy Memorial Endowment Fund for Biomedical Research (66992 to LS), the Claude Pepper Older Americans Independence Center (P30 AG024832 to EV), and the Sealy Center on Aging (grant to LS). MC was funded by the Onassis Foundation. SL is funded by a Canadian Institute of Health Research postdoctoral fellowship. CP was supported in part by a National Institute of Disability and Rehabilitation Research Postdoctoral Training Grant (H133P110012).

### Conflict of interest statement

The authors declare that the research was conducted in the absence of any commercial or financial relationships that could be construed as a potential conflict of interest.
